# Genetic Variation in Wheat Root Transcriptome Responses to Salinity: A Comparative Study of Tolerant and Sensitive Genotypes

**DOI:** 10.3390/ijms26010331

**Published:** 2025-01-02

**Authors:** Gang Wu, Xuelian Sun, Qingyi Sun, Xin Kang, Jiayan Wang, Xiaoyan He, Wenxing Liu, Dengan Xu, Xuehuan Dai, Wujun Ma, Jianbin Zeng

**Affiliations:** 1 College of Agronomy, Qingdao Agricultural University, Qingdao 266109, China; 20222201023@stu.qau.edu.cn (G.W.); 20232101043@stu.qau.edu.cn (X.S.); qingyisun0328@stu.qau.edu.cn (Q.S.); kangxin20041001@163.com (X.K.); wjy@stu.qau.edu.cn (J.W.); hexiaoyan@qau.edu.cn (X.H.); liuwx@qau.edu.cn (W.L.); xudengan@qau.edu.cn (D.X.); 202101034@qau.edu.cn (X.D.); 2 Academy of Dongying Efficient Agricultural Technology and Industry on Saline and Alkaline Land in Collaboration with Qingdao Agricultural University, Dongying 257347, China

**Keywords:** *Triticum aestivum*, RNA-Seq, differentially expressed genes (DEGs), salinity stress, molecular mechanisms

## Abstract

Salt tolerance is a critical trait for plant survival and productivity in saline environments. Development of salt tolerant crops is a practical strategy for addressing soil salinity issues. In this study, RNA-Seq analysis was performed using two wheat cultivars with contrasting salt tolerance (Neixiang188, tolerant and Barra, sensitive) at 6 h and 24 h after salinity treatment to determine the genetic variations reflected in the RNA expression patterns and identify key genes associated with salt tolerance. Our results revealed that there were 2983 upregulated and 1091 downregulated differentially expressed genes (DEGs), which were found in common in the two accessions. Meanwhile, 529 salt tolerant associated DEGs were subjected to GO function annotation, KEGG enrichment, and protein–protein interaction (PPI) network prediction. Finally, a theoretical framework outlining the salt tolerance mechanisms of Neixiang188 was proposed. It can be inferred that Neixiang188 possesses superior ion homeostasis, ROS detoxification, and osmotic adjustment abilities compared to Barra when subjected to saline stress. The present research sheds light on the genetic foundation of salt tolerance in wheat and offers candidate genes for genetic manipulation. Our research insights enhance the comprehension of the molecular mechanisms underlying salt stress responses and could guide future breeding efforts for improving salt tolerance in crops.

## 1. Introduction

Soil salinity is one of the largest global challenges limiting plant growth and crop productivity, as well as the increase in the demand for food crops [1]. Salt stress impacts over 800 million hectares of land, which represents 6% of the global land area, 20% of all arable land, and 33% of the world’s irrigated farmlands [1,2]. It has been aggravated because of the application of irrigation water with high salinity levels, climate change, and the increasing population [3]. Thus, salinity is becoming prominent and is an increasingly severe global problem that hampers agricultural productivity worldwide. To adapt to soil salinity, plants have developed a wide range of adaptations and mitigation strategies to balance growth and stress responses efficiently [4]. However, salinity tolerance is a sophisticated genetic trait that is largely determined by the intricate interplay between environmental and genetic factors, which remains partially elucidated [5]. Therefore, it is essential to unveil the underlying mechanisms of salinity tolerance in plants.

Due to the complex genetic nature of salt tolerance, the success of conventional breeding is very limited. Recent developments in omics technologies, including genomics, transcriptomics, and metabolomics, can assist in pinpointing crucial genes and biomolecules associated with salt tolerance [6]. Among them, transcriptomics is capable of uncovering key transcripts related to stress, the architecture of gene transcription, the functional pathways they are involved in, and various post-transcriptional modifications [7]. The RNA-seq based transcriptome approach is becoming more popular due to its significantly improved accuracy in quantifying transcript levels and its capability to identify transcript isoforms [7]. The high-throughput methods provide enhanced insights into crop tolerance to salinity stress. Several genes that could enhance salt tolerance were identified by the transcriptome analysis of ‘Sea Rice 86’ [8]. Chandran et al. (2019) identified 447 DEGs by employing RNA-seq for transcriptomic profiling in the japonica rice variety ‘Chilbounder’ under both stressed and non-stressed conditions [9]. Later, Cartagena et al. (2021) performed RNA sequencing to examine the root transcriptomes of salt-tolerant and salt-sensitive rice cultivars in response to saline stress [10]. In maize, a reasonable number of transcriptomics studies are available. A total of 1661 DEGs were detected in comparisons between the control and salinity stressed sample of B73 seedling leaves [11]. Wang et al. (2019) reported that 1856 upregulated DEGs were specific to salt-tolerant maize inbred line (L87) [12]. In addition, a series of advances in the salt tolerance of other crops was revealed by RNA seq technology in recent years.

Common wheat (*Triticum aestivum* L.), the most extensively cultivated crop globally and a significant cereal, accounts for approximately 20% of the calories and protein consumed daily in human diets [13,14,15]. As is well known, wheat is a crop with moderate salt tolerance that can experience a reduced yield when subjected to salt stress. Thus, for safeguarding food security, it is vital to explore salt-tolerance genes from common wheat germplasm and apply them to breeding. In the case of common wheat, with its vast and intricate hexaploid genome, the RNA-Seq technique serves as a rapid and effective method for mining candidate genes associated with salt tolerance [14]. The two most well-known and extensively studied salt tolerance pathways in wheat are the sodium ion exclusion pathway mediated by the high-affinity K^+^ transporter (HKT) and the ROS homeostasis mechanism mediated by SIMILAR to RCD ONE (SRO) [16]. Over the past few years, several salt-tolerance genes in wheat have been successively identified. For instance, *TmHKT1;5* is a candidate gene for *Nax2*, and by introgressing *TmHKT1;5-A* into common tetraploid wheat through hybridization, the yield can be increased by 25% in saline–alkali soils [17]. The wheat-specific allele *TaSPL6-DIn* enhances salt tolerance in wheat by activating *TaHKT1;5-D* without affecting yield-related traits [18]. Additionally, the promoter of the wheat *TaCHP* gene contains multiple SNPs and Indels, forming two haplotypes, Hap1 and Hap2, among which accessions carrying Hap1 exhibit significantly higher salt tolerance and *TaCHP* expression levels than those carrying Hap2 [19]. Furthermore, it was reported that TaSRO1 interacting protein, SIP (*TaSIP1*), a member of the NAC transcription factors family, is capable of interacting with the TaSRO1 protein, thereby modulating salt tolerance in wheat [20].

In this study, RNA-seq analysis was conducted on the roots of a salt-tolerant and a salt-sensitive wheat germplasm. The aim was to identify key salt-tolerance genes in wheat, exploring the genetic differences at the transcriptional level, and providing genetic resources for functional identification of salt-tolerance genes and the breeding of new salt-tolerant wheat varieties.

## 2. Results

### 2.1. The Phenotypic Differences Between Neixiang188 and Barra After Salt Treatment

**Figure 1:**
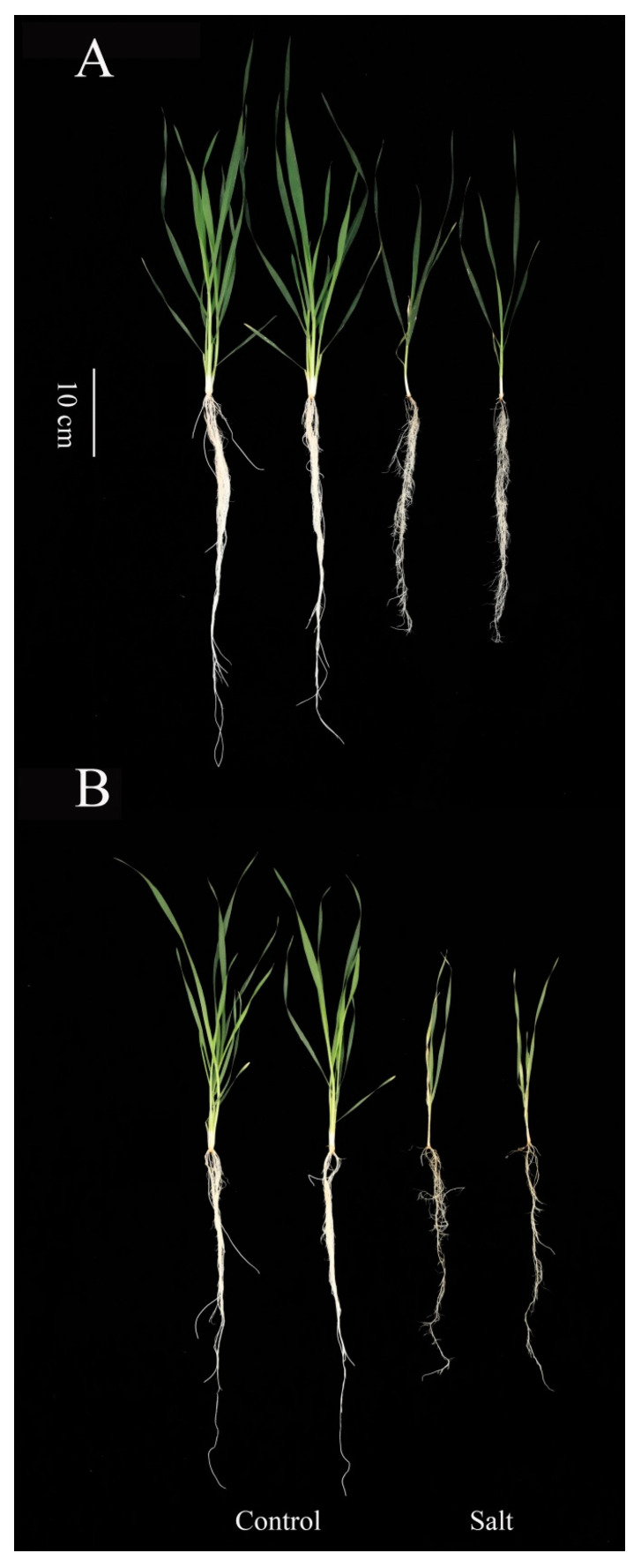
Comparison of the morphological changes of different wheat varieties under salinity stress conditions. The phenotypes of (**A**) Neixaing188 and (**B**) Barra were evaluated after 15 days of salinity treatment. Bar = 100 mm.

**Figure 2:**
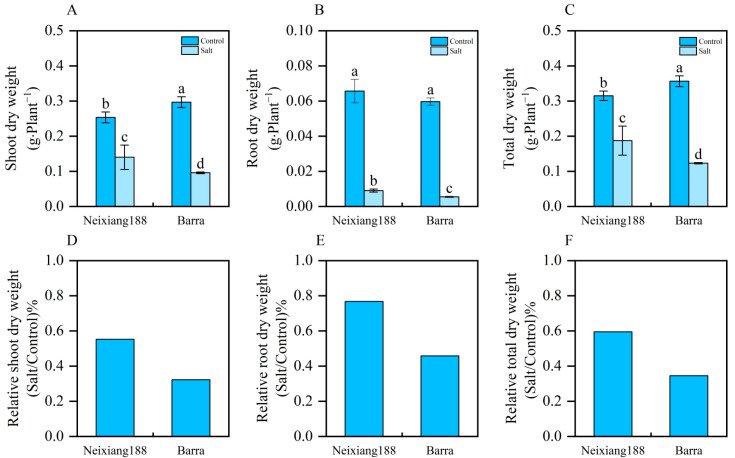
Phenotypes of wheat seedlings under normal and salinity stress conditions. (**A**) Shoot dry weight. (**B**) Root dry weight. (**C**) Total dry weight. (**D**) Relative shoot dry weight. (**E**) Relative root dry weight. (**F**) Relative total dry weight. The different letters represent significant differences according to Duncan’s multiple range, *p* < 0.05, *n* = 4.

After 15 days of treatment with 200 mM NaCl, the growth of both Neixiang188 and Barra was significantly inhibited (Figure 1). However, the more visible symptoms of salinity stress were observed in Barra (Figure 1). The degree of yellowing in old leaves was more severe in Barra in addition to weak plants (Figure 1). Relative to the control group, the salinity treatment led to a marked decrease in dry weight for both the shoots and roots across the two genotypes (Figure 2). For shoot dry weight, both Neixiang188 and Barra showed significant reductions, being 67.8% and 44.7%, respectively (Figure 2). There was little difference in root dry weight between Neixiang188 and Barra under normal conditions, while the root dry weight of Neixiang188 was higher than that of Barra in response to salinity stress, being 1.6 times larger (Figure 2). Clearly, the current results indicate that Neixiang188 is salt-tolerant, while Barra is salt-sensitive.

### 2.2. Screening of DEGs

**Figure 3:**
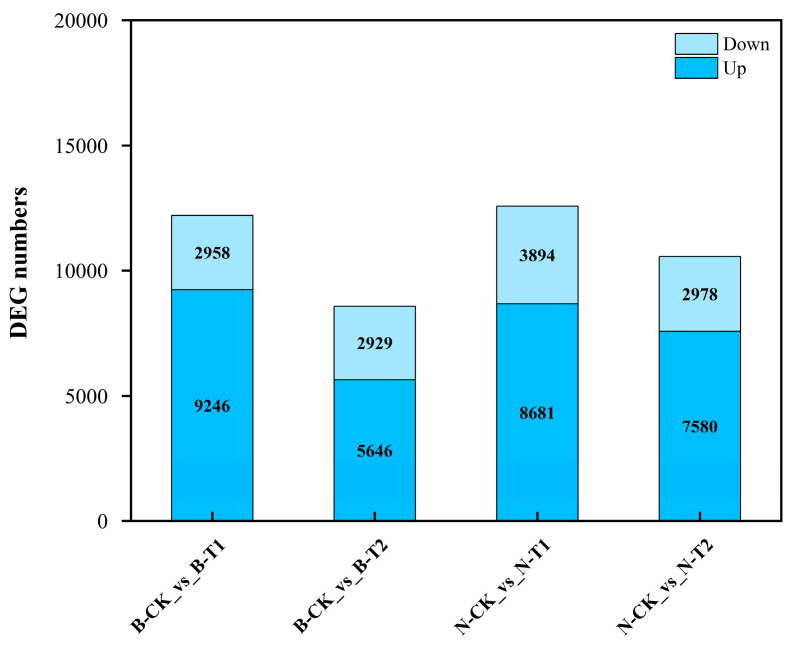
Numbers of DEGs in wheat during salinity stress. Note: B, Barra; CK, Control; T1, Treatment for 6 h; T2, Treatment for 24 h; N, Neixiang188.

**Figure 4:**
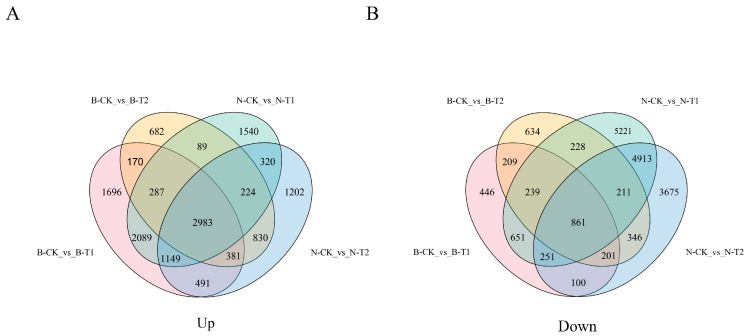
Venn diagrams of DEGs in response to salinity stress in Neixiang188 and Barra. Venn diagrams of upregulated (**A**) and downregulated (**B**) genes at 6 h and 24 h after the initiation of salinity treatment. Note: B, Barra; CK, Control; T1, Treatment for 6 h; T2, Treatment for 24 h; N, Neixiang188.

For RNA-Seq analysis, we developed a total of eighteen cDNA libraries derived from two genotypes after saline treatment for the indicated times (6 h and 24 h). We obtained raw reads ranging from 41,728,952 to 58,966,734 (Supplemental Table S1). The percentages of clean reads aligned to the reference genome were 91.64–94.53%, with 3.61–4.75% being multiple mapped clean reads and 88.17–90.38% being uniquely mapped clean reads (Supplemental Table S1). The total numbers of genes with expression detection were 71,376 (Supplemental Table S2). Then, DEGs were identified based on their fold change and false discovery rate (FDR). A total of 12,575 DEGs were obtained at 6 h under salinity stress in Neixiang188 accession, with 8681 upregulated and 3894 downregulated, respectively (Figure 3). Nearly the same amount of DEGs were detected at 6 h in Barra, including 9246 upregulated and 2958 downregulated (Figure 3). The number of DEGs in Neixiang188 was almost 1.23 times as large as that of Barra at 24 h, being 7580 and 5646 upregulated, respectively (Figure 3). There were 2983 upregulated (Figure 4A) and 1091 downregulated DEGs (Figure 4B), which were found in common in the two accessions.

In the current study, 657 transcription factors (TFs) whose expression level was either upregulated or downregulated in at least one comparison group were identified (Supplemental Table S2), and they belonged to various families, including *MYB* (366), *WRKY* (108), *bHLH* (83), *ERF* (58), *bZIP* (37), *MADS-box* (23), etc. (Supplemental Table S2). Among them, we found that *MYB* were the most enriched, accounting for 55.7% of all TFs. Among them, the expression levels of genes *TraesCS3A03G0090300* (*MYB21*), *TraesCS3D03G0065300* (*MYB108-like*), and *TraesCS4B03G0504600* (*MYB86-like*) in Neixaing188 were higher than in Barra (Supplemental Table S3). In general, most TFs responded to saline stress rapidly and then returned to their initial expression levels, while a small subset exhibited sustained expression or were induced only after 24 h of NaCl treatment. 

Meanwhile, we also focused on DEGs encoding ion transporters, which are probably involved in ion homeostasis under salinity stress. Interestingly, 27 DEGs encoding potassium transporters were identified, most of which were upregulated at 6 h or 24 h after salt treatment (Supplemental Table S3). Under salt stress, the gene *TraesCS2D03G0226300* (*HAK9*) was upregulated in Neixaing188 by approximately twice as much as in Barra. Similar expression trends were observed in transporter genes TraesCS1803G0845600 (ZIP5 Zine transporter), *TraesCS5B03G0470600* (*TPKC*), *TraesCS7403G0212600* (*SULTR3;4*), and TraesCS7D03G0192800 (*SULTR3;4*). In addition, DEGs involved in oxidative stress were also investigated, such as oxidoreductase, glutathione S-transferase, and peroxidase, and they play a crucial role in controlling ROS generation and mitigating cellular damage in response to salt stress (Supplemental Table S3). For instance, *TraesCS1A03G0823100 (SRGI)* and the proteins encoded by the genes *TraesCS1D03G0263900*, *TraesCS3.403G1261200*, and *TraesCS5403G1158400* possess oxidoreductase activity (Supplemental Table S3).

### 2.3. GO Functional Annotation and Enrichment Analysis of DEGs

GO functional annotation and enrichment analysis were performed for the DEGs obtained by comparing the control and treated groups pairwise between Neixiang188 and Barra (Supplemental Figures S1 and S2). The cellular processes (GO:0009987) and metabolic processes (GO:0008152) were ranked in the top two in terms of biological processes category, while binding (GO:0005488) and catalytic activity (GO:0003824) were the dominant functional groups for molecular function category (Supplemental Figure S1). At 6 h after treatment, the DEGs response to oxidative stress and response to chemical were enriched in Neixaing188, while carbohydrate metabolic processes and response to chemical occupied the largest share in Barra (Supplemental Figure S2). However, carbohydrate metabolic processes and response to oxidative stress were both enriched in the two genotypes at 24 h (Supplemental Figure S2).

**Figure 5:**
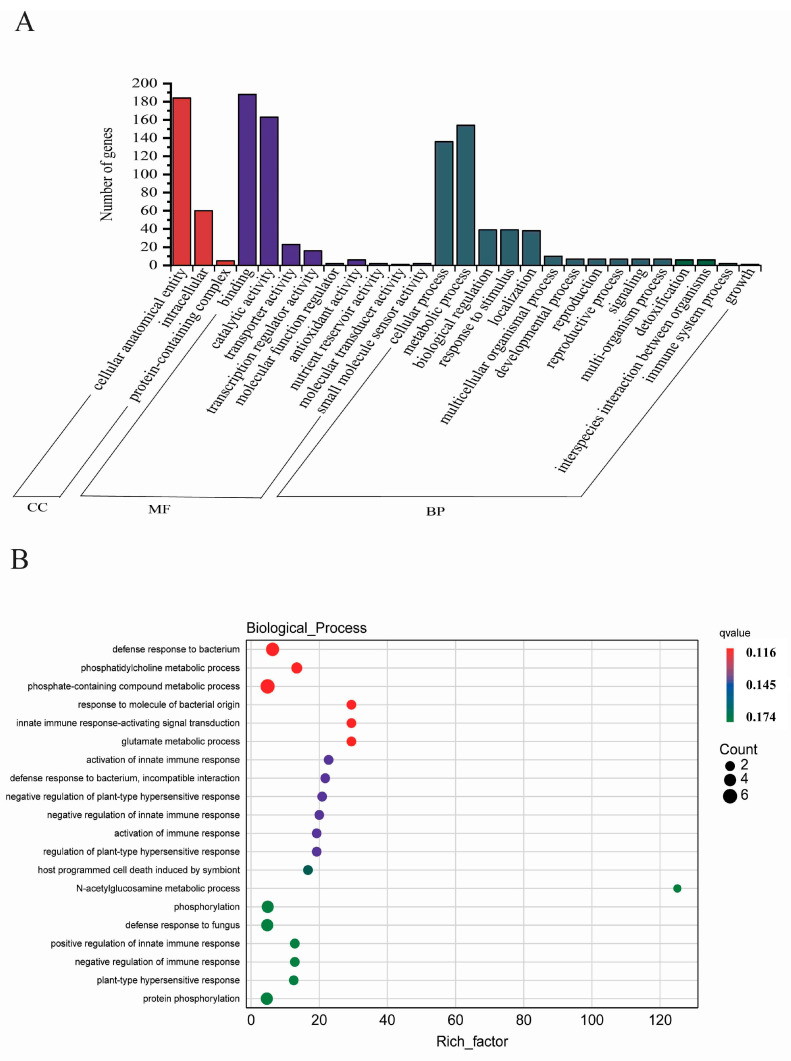
Distribution of the functional GO categories of the 529 DEGs in wheat. (**A**) GO classification of the 529 DEGs. CC, cellular component; BP, biological process; and MF, molecular function. (**B**) Statistics of GO enrichment. Rich factor is an indicator that measures the degree of gene enrichment, reflecting the ratio of the number of DEGs to the total number of genes annotated to the pathway. The larger this ratio, the greater the enrichment of differential genes in that metabolic pathway or functional category.

In our research, we concentrated on DEGs that showed a marked increase in expression in the roots of Neixiang188, yet were either decreased or remained stable in Barra, or were stable in Neixiang188 but decreased in Barra. A total of 529 DEGs fitting these criteria were identified and subjected to further analysis (Supplemental Table S4). From the perspectives of cellular components, molecular functions, and biological processes, the above 529 DEGs were annotated with GO functions, comprising 3, 9, and 16 secondary GO term categories, respectively (Figure 5A). It is noteworthy that genes annotated as cellular anatomical entity (GO:0110165) and intracellular (GO:0005622) accounted for 73% and 24% of the cellular components category, respectively. Within the molecular functions category, the predominant functional groups were binding and catalytic activity, representing 46% and 40% of the DEGs, respectively (Figure 5A). Within the biological processes category, the metabolic processes and cellular processes emerged as the dominant functional groups, representing 33% and 29% of the DEGs, respectively (Figure 5A). The GO enrichment analysis for DEGs showed that 12 of the top 20 most significantly enriched categories were associated with molecular functions. Seven were related to cellular components, and one was related to biological processes (Figure 5B). In the molecular function category, DEGs were predominantly associated with three GO terms: ATP binding (GO:0005524), DNA binding (GO:0003677), and protein kinase activity (GO:0004672). The ATP binding GO term showed the highest level of enrichment, encompassing 21% of the genes within the molecular function category (Figure 5B). In the cellular components category, a total of 133 DEGs were annotated to this category, with the most enrichment observed in the nucleus (GO:0005634) GO term, accounting for 27% of the DEGs enriched in this category (Figure 5B). In the biological processes category, enrichment was only observed in one GO term (GO:0008150).

### 2.4. The KEGG Enrichment Results of DEGs

KEGG classification and enrichment analysis were also performed for all differential genes obtained by comparing the control and treated groups pairwise between Neixiang188 and Barra (Supplemental Figures S3 and S4). At 6 h after salt treatment, the numbers of DEGs categorized into five main groups ranged from 40 to 520, while the number of DEGs involved in the most highly represented KEGG categories was 299 (Supplemental Figure S3). After KEGG enrichment analysis, it was found that there are over 200 DEGs involved in phenylpropanoid biosynthesis in the two accessions (Supplemental Figure S4).

**Figure 6:**
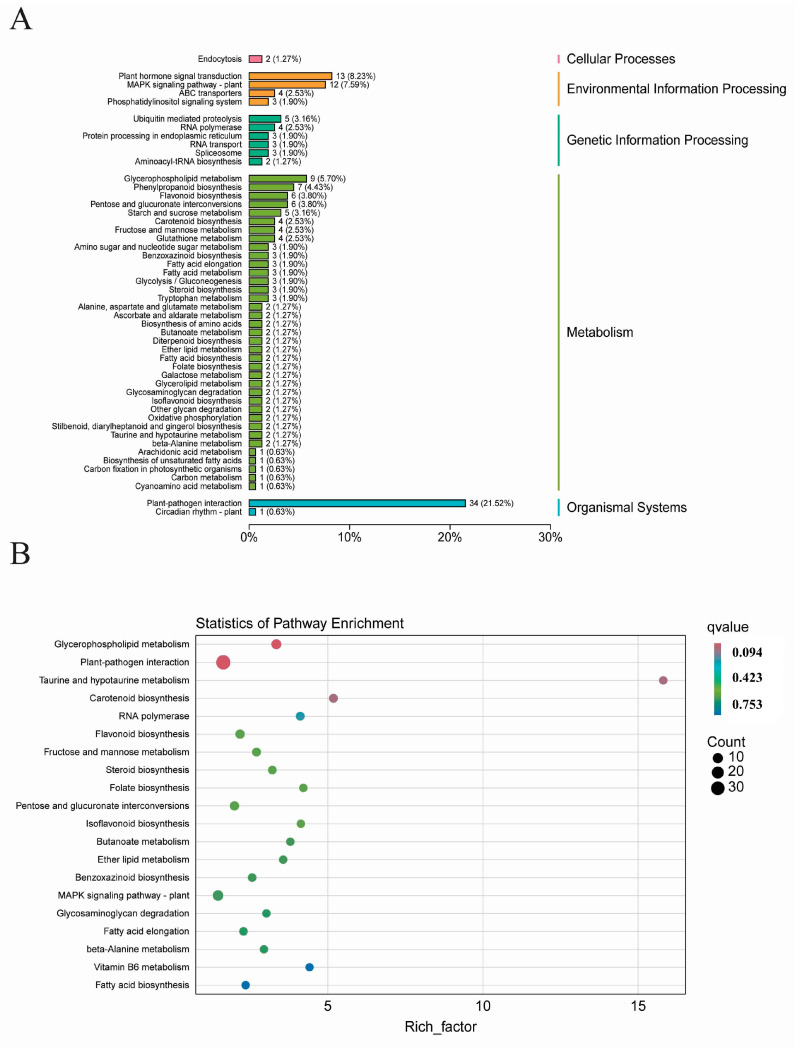
Distribution of the KEGG categories of the 529 DEGs in wheat. (**A**) KEGG classification of the 352 DEGs. (**B**) Statistics of KEGG enrichment. Rich factor is an indicator that measures the degree of gene enrichment, reflecting the ratio of the number of DEGs to the total number of genes annotated to the pathway. The larger this ratio, the greater the enrichment of differential genes in that metabolic pathway or functional category.

The KEGG analysis of the 529 DEGs revealed that these genes could be categorized into five main groups: cellular processes (2), genetic information processing (32), environmental information processing (20), metabolism (105), and organismal systems (35) (Figure 6A). Among these pathways, the plant-pathogen interaction (ko04626) pathway had the largest number of annotated DEGs, totaling 34 genes (Figure 6B). Following closely behind was the hormone signal transduction pathway and the phenylpropanoid biosynthesis pathway, accounting for 5.9%, indicating these two metabolic pathways play a vital role in stress resistance.

The top 20 KEGGs were significantly enriched by means of KEGG enrichment analysis (Figure 6B). Plant–pathogen interaction (ko04626), plant hormone signal transduction (ko04075), MAPK signaling pathway—plant (ko04016), and glycerophospholipid metabolism (ko00564) showed greater enrichment significance in comparison to other KEGG pathways, with rich factor values of 21.5% (34 out of 158), 8.22% (13 out of 158), 7.59% (12 out of 158), and 5.69% (9 out of 158), respectively (Figure 6B).

### 2.5. The PPI Network Analysis of RNA Expression Profile

**Figure 7:**
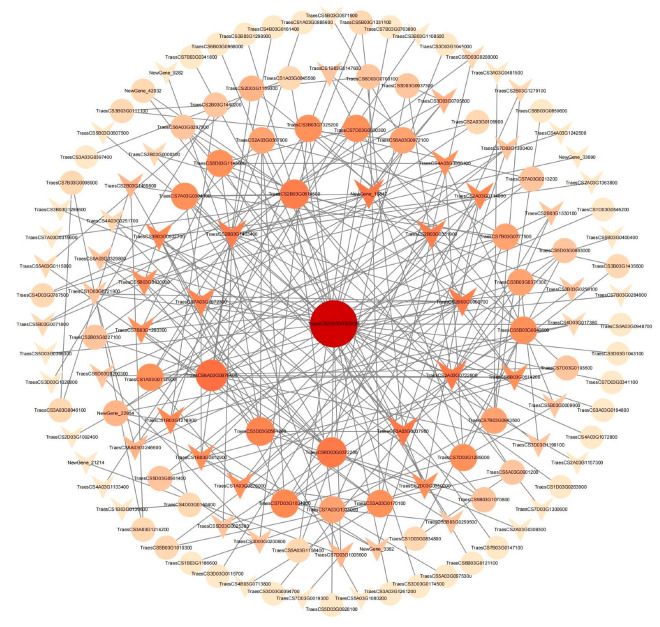
Protein–protein interactions (PPI) of the 529 DEGs in the two wheat genotypes. Circles represent upregulated genes, and triangles represent downregulated genes. Larger circles and triangles indicate genes with more interacting proteins.

By leveraging the STRING database, we forecasted the protein–protein interaction (PPI) network for 529 DEGs and uncovered a total of 251 interacting pairs (Figure 7). The results showed that the upregulated genes *TraesCS2D03G0323600* (1-Cys peroxiredoxin PER), *TraesCS6A03G0976400* (haloacid dehalogenase-like hydrolase domain-containing protein), *TraesCS6D03G0372200* (ADP, ATP carrier protein 1, chloroplastic), and *TraesCS2B03G0514500* (interferon-related developmental regulator 1) had a relatively high number of interacting proteins, with 33, 12, 10, and 9 interactions, respectively (Figure 7). Among the downregulated genes, *TraesCS2A03G0722800* (Nudix hydrolase 12, mitochondria), *NewGene_10847* (NMalate dehydrogenase NADP 1), *TraesCS3A03G0037900* (rust resistance kinase Lr10)*,* had the highest number of interacting proteins, totaling 10 (Figure 7). In addition, the outermost layer of the graph is distributed with gene types that have a relatively low number of interacting pairs, generally fewer than three.

### 2.6. qRT-PCR Confirm the Expression Level of the DEGs

**Figure 8:**
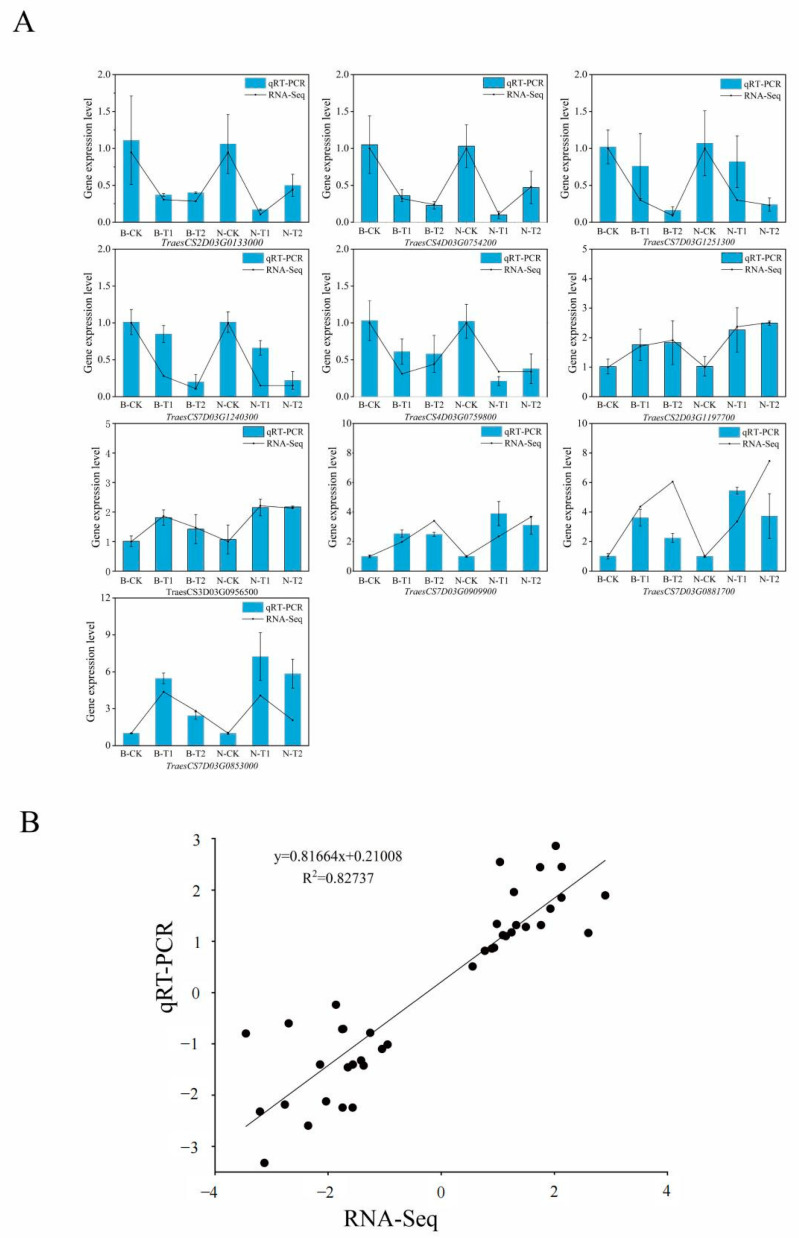
Quantitative real-time PCR (qRT-PCR) confirmation of the transcriptomic profiles of selected genes. (**A**) The gene expression level in RNA-Seq data of ten tested genes compared with real-time PCR results. (**B**) The correlation coefficient was determined between RNA-seq and RT-PCR data. Note: B, Barra; CK, Control; T1, Treatment for 6 h; T2, Treatment for 24 h; N, Neixiang188. Sequences of gene specific primers used for PCR are listed in Supplemental Table S5.

To confirm the RNA-Seq data indicating changes in transcript levels, the expression levels of ten DEGs in wheat roots were evaluated using quantitative real-time PCR, including *TraesCS7D03G1240300* (polyamine oxidase 1), *TraesCS4D03G0759800* (serine acetyltransferase), *TraesCS7D03G1251300* (rhodanese-like domain-containing protein 15), *TraesCS7D03G0881700* (CBL-interacting protein kinase 24), *TraesCS7D03G0874000* (O-fucosyltransferase 8), *TraesCS7D03G0909900* (Nudix hydrolase 2), *TraesCS2D03G0133000* (transcription factor RAX2-like), *TraesCS2D03G1197700* (bHLH112-like), *TraesCS3D03G0956500* (auxin response factor 4-like), and *TraesCS4D03G0754200* (metal tolerance protein 1-like). The results showed that over a 2-fold increase in expression level were observed for *TraesCS2D03G1197700*, *TraesCS7D03G0909900*, *TraesCS7D03G0881700*, and *TraesCS7D03G0853000* in Neixiang188, being much higher than in Barra (Figure 8A). Conversely, the expression levels of the two genes *TraesCS2D03G0133000* and *TraesCS4D03G0754200* were downregulated in both genotypes, but Barra showed a greater reduction compared to Neixiang188 at 24 h of salinity stress (Figure 8A). In addition, *TraesCS7D03G1240300* and *TraesCS4D03G0759800* were downregulated in both genotypes, but Barra showed a smaller decrease compared to Neixiang188. The correlation coefficient was determined between the RNA-seq and RT-PCR data based on the outcomes of ten genes. The R^2^ value was greater than 0.8, indicating the reliability of the RNA-seq results (Figure 8B).

## 3. Discussion

As critical factors, transcription factors (TFs) are regulators that target specific DNA regulatory elements directly for activating or repressing the expression of their downstream genes [21]. TFs are crucial in modulating salinity responses by regulating genes involved in the detection and transmission of salt-stress signals, as well as genes related to salt functions such as ion transport, osmotic adjustment, and oxidative stress management [22,23,24]. For instance, it was reported that *OsMYB106* bound to the MYB binding cis-element (MYBE) in the *OsHKT1;5* promoter, thereby activating *OsHKT1;5* expression during salinity stress [25]. Zhang et al. (2020) found that heterologously expressing *GmMYB84* in *Arabidopsis thaliana* plants resulted in higher germination rates and more tolerance to salt stress than the wild type [26]. *OsWRKY54* was identified as a key regulator in salt tolerance, as it binds to the W-box element in the promoter region of *OsHKT1;5* [27]. Another study by Lu et al. (2023) in Arabidopsis found that *CycC1;1* physically associates with *WRKY75*, a transcription factor capable of binding to the *SOS1* promoter, thereby enhancing *SOS1* expression and modulating salt tolerance [28]. Similarly, several transcription factors associated with the salt stress response have been discovered in wheat, such as those from the *MYB*, *bHLH*, *ERF* and other family member genes, such as *R2R3-MYB* transcription factor *TaSIM*, *TabHLH1*, *TaNAC29*, *TaWRKY93*, *TaNF-YA10-1*, *TaMYB73*, and *TaSPL6-D* [18,29,30,31,32,33,34]. In this study, we discovered more than a hundred transcription factors encompassing various families such as *AP2/ERF*, *bZIP*, *MYB*, *bHLH*, *NAC*, *Zn-finger*, and *G2-like* that showed altered expression under salinity stress, more than 10 of which were highly expressed in the tolerant variety. Considering their crucial role in enhancing stress tolerance, they are considered to be key targets in genetic manipulation for dissecting the molecular mechanisms underlying salt tolerance.

**Figure 9:**
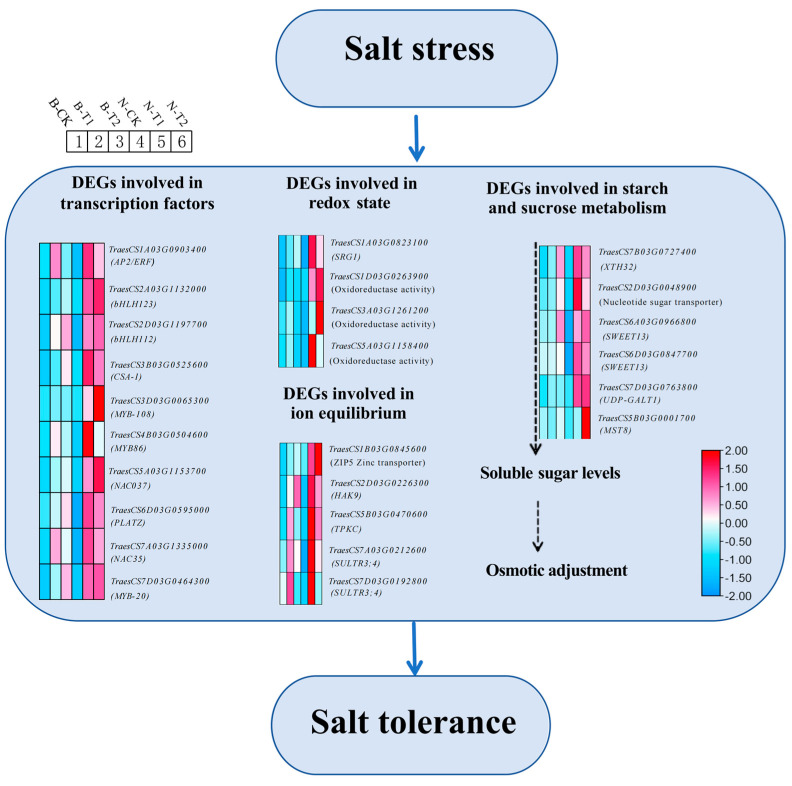
A hypothetical model of the mechanism involved in salinity stress tolerance in Neixiang188. The relative expression levels of the key DEGs are shown by a color gradient from low (blue) to high (red). The column of each heatmap from left to right: Barra—Control, Barra—6 h, Barra—24 h, Neixiang188—Control, Neixiang188—6 h, Neixiang188—24 h.

Soil salinity is regarded as a severely damaging challenge to plant growth and yield. It inflicts both osmotic stress and ion toxicity, adversely affecting key processes such as plant nutrition, cellular metabolism, and photosynthesis [2]. A primary impact of salinity stress is the disruption of ion balance across plant tissues [2]. It has been noted that salinity stress leads to a significant accumulation of Na^+^ and a reduction in K^+^, resulting in ionic imbalance [35]. Thus, maintaining a high cytosolic K^+^/Na^+^ ratio is crucial for plants under salinity stress. Intensive studies have been conducted on Na^+^ influx, extrusion and K^+^ uptake mechanisms, and numerous ion channels and transporters, such as PM-H^+^ATPase, V-H^+^ATPase, CNGC, HKTs, HAK/KUP/KT, and NHXs, have been identified [35]. Among them, the HKT proteins are categorized into two subgroups based on their transport specificity, attracting significant attention due to certain members within this group that are crucial for plants’ salt tolerance [35]. For instance, transgenic plants that overexpress *AtHKT1;1* in their root stele cells exhibit reduced salt accumulation in the shoots and enhanced salt tolerance [36]. *OsHKT1;5* was found to be involved in limiting Na^+^ transport to young rice leaves, whereas the role of *OsHKT1;5* was proposed to mainly mediate Na^+^ influx into roots [37]. It was revealed that transgenic vegetable plants with the *TaNHX2* gene added through a genetic engineering approach were tolerant to salinity stress [38]. It was demonstrated by Shen et al. (2015) that *OsHAK21* plays crucial roles in the maintenance of the Na^+^/K^+^ homeostasis by mediating K^+^ absorption in rice under salt stress [39]. Moreover, the excessive accumulation of Na^+^ in the root zone also disrupts the absorption of essential nutrients such as calcium, nitrogen, sulfur, and potassium, leading to an imbalance in nutrient uptake [2,35]. In the current study, we identified several important transporter-encoding genes, such as *TraesCS1B03G0845600* (*ZIP5* Zinc transporter), *TraesCS2D03G0226300* (*HAK9*), and *TraesCS5B03G0470600* (*TPKC*), which exhibit markedly elevated expression in genotypes with higher tolerance compared to those that are more sensitive (Figure 9, Supplemental Table S3). Hence, it can be inferred that the robust ion balance ability of the salt-tolerant genotypes constitutes one of the mechanisms that confer their salt tolerance.

Apart from osmotic stress, ionic stress, and nutrient imbalance, plants frequently encounter increased production of reactive oxygen species (ROS), resulting in oxidative stress in saline conditions [40,41]. Overabundance of ROS triggers phytotoxic effects like protein breakdown, enzyme deactivation, lipid peroxidation, and DNA denaturation [42]. To counteract ROS-induced damage, plants have developed a complex antioxidant defense system comprising both enzymatic and non-enzymatic components [42]. Well-studied key antioxidants in plant cells help detoxify reactive oxygen species (ROS) in plant cells under abiotic stresses, including salinity stress [40,43]. Up to now, the genes involving ROS homeostasis have been extensively studied, and their potential applications in improving salt tolerance have been evaluated [40]. For instance, expression of the soybean *GmPAP3* gene is upregulated by salt stress, and its ectopic expression in transgenic tobacco and *Arabidopsis thaliana* mimics the protective role of the antioxidant ascorbic acid [43]. It was found that *OsOPR7* was localized in the peroxisomes and its overexpression in seedlings confers high tolerance to salinity stress [44]. Furthermore, *TaSRO1* influences the expression of numerous genes related to ROS homeostasis, which is considered a key mechanism for salt tolerance in wheat [20,43]. In the current study, the expression of the antioxidant-related gene *SRG1* was significantly higher in the tolerant genotype Neixiang188 compared to the sensitive genotype Barra (Figure 9, Supplemental Table S3). Meanwhile, three DEGs encoding oxidoreductase were upregulated in Neixiang188, while there was little change in Barra (Figure 9, Supplemental Table S3). In this context, it may be assumed that the tolerant genotype Neixiang188 possesses strong antioxidant capabilities under salinity stress, which can effectively eliminate excessive ROS and reduce the extent of damage, thereby exhibiting strong salt tolerance.

Furthermore, we also discovered several genes associated with the metabolic pathways of starch and sucrose. For instance, genes such as *TraesCS7B03G0727400* (*XTH32*), *TraesCS7D03G0763800* (*UDP-GALT1*) and *TraesCS6A03G0966800* (*SWEET13*) were expressed at higher levels in the tolerant genotypes compared to the sensitive genotypes, which were associated with the content of soluble sugars (Figure 9, Supplemental Table S3). At the same time, the roles of compatible solutes in salinity stress tolerance have been studied extensively [45,46]. Thus, it may be assumed that disparities in osmotic regulation abilities also account for the variance in salt tolerance observed between the two genotypes.

## 4. Materials and Methods

### 4.1. Plant Materials and Salt Treatment

Two common wheat accessions, Neixiang188 (salt-tolerant, hexaploid, China) and Barra (salt-sensitive, hexaploid, Italy) cultivars were obtained from Institute of Crop Sciences, Chinese Academy of Agricultural Sciences (CAAS), China [47]. Seeds were sterilized by immersing in 2% H_2_O_2_ for 30 min. Subsequently, they were rinsed three times with distilled water prior to being placed in germination boxes lined with moistened filter paper for sprouting. After 10 days of germination, the uniform germinated seedlings were then transferred to plastic pots (10 L) for hydroponic incubation. The hydroponic experiment was performed in a greenhouse (22 °C/18 °C, 16 h/8 h, day/night), with aerated hydroponic solution containing 1 mM Ca(NO_3_)_2_.4H_2_O, 1 mM KNO_3_, 0.4 mM MgSO_4_.7H_2_O, 0.2 mM NH_4_H_2_PO_4_, 0.5 μM MnCl_2_.4H_2_O, 3 μM H_3_BO_3_, 0.5 μM (NH_4_)_6_Mo_7_O_24_, 0.2 μM CuSO_4_.5H_2_O, 0.3 μM ZnSO_4_.7H_2_O, and 0.2 mM Fe(III)-EDTA.

Plants were grown in the nutrient solution at half strength for a week, then transitioned to a full-strength solution for an additional week, with the solution being replaced every three days. Ten-day-old seedlings that had grown to the three-leaf stage were subsequently subjected to saline stress [48]. The treatment group was subjected to salt stress in the form of NaCl, with a final concentration of 200 mM, and the nutrient solution without NaCl as a control.

### 4.2. Biomass Identification

After 15 days of salt treatment, the roots of all plants were washed with distilled water and dried using paper towels. Subsequently, the roots and shoots of the seedlings were detached and collected for analysis, with four biological replicates. All plant specimens were put in an oven and dehydrated at 80 °C to achieve a stable weight for dry weight assessment. The relative dry weight was calculated as the proportion of the dry weight under treatment conditions to that under control conditions.

### 4.3. RNA Extraction, cDNA Library Construction and RNA-Sequencing

For RNA-Seq analysis, seeds of Neixiang188 and Barra were germinated under identical conditions in a hydroponic setup within a plant growth chamber. Seedlings at the two-leaf stage were then subjected to salt stress using a 200 mM NaCl solution for durations of 6 h and 24 h, respectively. Root samples from both control and salt-stressed plants were gathered in triplicate. The samples were rapidly frozen in liquid nitrogen for later RNA sequencing, with three biological replicates. RNA was extracted using the SteadyDrop Plant RNA Extraction Kit (Accurate Biology, Qingdao, China) as per the manufacturer’s instructions. The RNA’s purity and concentration were assessed by NanoDrop 2000 (Thermo, Wilmington, NC, USA).

mRNA was extracted using magnetic beads coated with oligo (dT) and then fragmented with a buffer solution. Subsequently, the mRNA served as a template for the synthesis of double-stranded cDNA, which included adapters on both ends. The cDNA libraries were constructed by amplification of adapter sequences, quantity was measured with a Qubit 2.0 fluorometer, and the size of the insert fragments was verified using an Agilent 2100 Bioanalyzer (Santa Clara, CA, USA). qRT-PCR was also performed for accurate quantification and evaluating library quality.

Then, the qualified complementary DNA (cDNA) libraries underwent paired-end sequencing with a read length of 2 × 150 base pairs on the Illumina HiSeq 2500 platform (San Diego, CA, USA) to generate raw sequence data. Sequence reads that included adapter sequences and those identified as low quality were removed (N content > 10% and Q ≤ 20) using Fastp (v0.23.4) [49]. HISAT was employed to align quality-controlled reads to the existing wheat genome (Triticum aestivum.v2.1.genome.fa) and structural annotations [49].

### 4.4. Identification of the Differentially Expression Genes (DEGs)

Gene expression levels were quantified based on the number of fragments per kilobase of transcript per million mapped fragments (FPKM). The read comparison process was performed by featureCounts program [50]. Analysis of differential gene expression across samples was performed utilizing the DESeq2 package (v1.46.0). Following this, the FDR was calculated by applying the Benjamini–Hochberg procedure to adjust the *p*-values from the hypothesis tests [51,52]. The final list of DEGs was obtained based on the filtering criteria of |log_2_^FoldChange^| > 1, FDR < 0.01, and FPKM ≥ 1.

### 4.5. Functional Annotation, GO Enrichment and KEGG Analysis

WEGO software (v2.0) was employed to categorize the Gene Ontology (GO) functions of DEGs across biological processes (BP), molecular functions (MF), and cellular components (CC). GO enrichment for DEGs was conducted using the singular enrichment analysis (SEA) approach with a significance level of *p* < 0.01 and FDR less than 0.05, as implemented in AgriGO. Additionally, the KEGG Orthology Based Annotation System was utilized to assess the statistical overrepresentation of DEGs in the KEGG pathways, with a significance threshold set at *p* value < 0.01 and FDR < 0.05.

### 4.6. Protein–Protein Interaction (PPI) Networks

The protein sequences associated with the selected DEGs were submitted and uploaded to the STRING database for analysis. Wheat was set as the default species for analysis, and the combined score threshold was established at above 0.6. Then, we conducted a study on protein–protein interactions. Finally, the results of the PPI networks were presented through the visualization software Cytoscape (v3.7.2).

### 4.7. Validation of RNA-Seq by Real-Time Quantitative Polymerase Chain Reaction (RT-qPCR)

To confirm the reliability of the RNA-Seq results, ten DEGs were randomly selected for PCR assays to evaluate the expression levels. Gene-specific primers were crafted using the primer-blast tool and subsequently underwent BLAST analysis against the Chinese Spring reference genome to verify their specificity (Supplemental Table S5). The *EF1a* gene was used as a housekeeping gene. Real-time PCR was performed using the QuantStudio™ 3 Real-Time PCR System from Thermo Fisher Scientific, following the protocol: initial denaturation at 95 °C for 30 s, then 40 cycles of denaturation at 95 °C for 5 s and annealing/extension at 60 °C for 30 s [53].

### 4.8. Statistical Analysis

The statistical analysis of physiological characteristics and gene expression data was conducted using IBM SPSS v24 software. A *p*-value below the threshold of 0.05 was regarded as statistically significant, and a *p*-value less than 0.01 was deemed highly significant.

## 5. Conclusions

The current results indicate that there are significant differences in transcription levels between the tolerant (Neixiang188) and sensitive (Barra) genotypes under salinity conditions. A total of 529 salt tolerant associated DEGs were identified and subjected to analyses of GO function annotation, KEGG enrichment, and PPI network prediction. Based on the available findings, the salt tolerance mechanism in Neixiang188 could be summarized by a hypothetical model (Figure 9). The DEGs, including transcription factors such as *TraesCS3A03G0090300* (*MYB21*), *TraesCS3D03G0065300* (*MYB108-like*), and *TraesCS4B03G0504600* (*MYB86-like*); DEGs such as *TraesCS2D03G0226300* (*HAK9*) involved in ion equilibrium; DEGs such as *TaesCS1403G0823100* (*SRGI*) involved in redox state; as well as the other DEGs shown in Figure 9 are all candidates for further attention. It is concluded that Neixiang188 possesses superior ion homeostasis, ROS detoxification, and osmotic adjustment abilities compared to Barra when subjected to salt stress. The identified genes are crucial for improving salt tolerance in wheat.

## Data Availability

The RNA-seq data has been deposited at the National Center for Biotechnology Information (NCBI) and the accession number is PRJNA1192705.

## Supplementary Materials

The supporting information can be downloaded at https://www.mdpi.com/article/10.3390/ijms26010331/s1.
