# Supplementary material for: Genetic Variation in Wheat Root Transcriptome Responses to Salinity: A Comparative Study of Tolerant and Sensitive Genotypes
Source: Int J Mol Sci. 2025 Jan 2;26(1):331. doi: 10.3390/ijms26010331 (PMC11720974; doi:10.3390/ijms26010331)
Supplement: Supplementary file 1 [file ijms-26-00331-s001.zip › Figure legends.pdf]

Supplemental Figure S1. Distribution of the functional GO categories of all DEGs in Neixiang188 and Barra.

Supplemental Figure S2. GO enrichment of all DEGs in Neixiang188 and Barra.

Supplemental Figure S3. KEGG classification of all DEGs in Neixiang188 and Barra.

Supplemental Figure S4. KEGG enrichment of all DEGs in Neixiang188 and Barra.
