# Supplementary figures and images for: Genetic Variation in Wheat Root Transcriptome Responses to Salinity: A Comparative Study of Tolerant and Sensitive Genotypes
Source: Int J Mol Sci. 2025 Jan 2;26(1):331. doi: 10.3390/ijms26010331 (PMC11720974; doi:10.3390/ijms26010331)

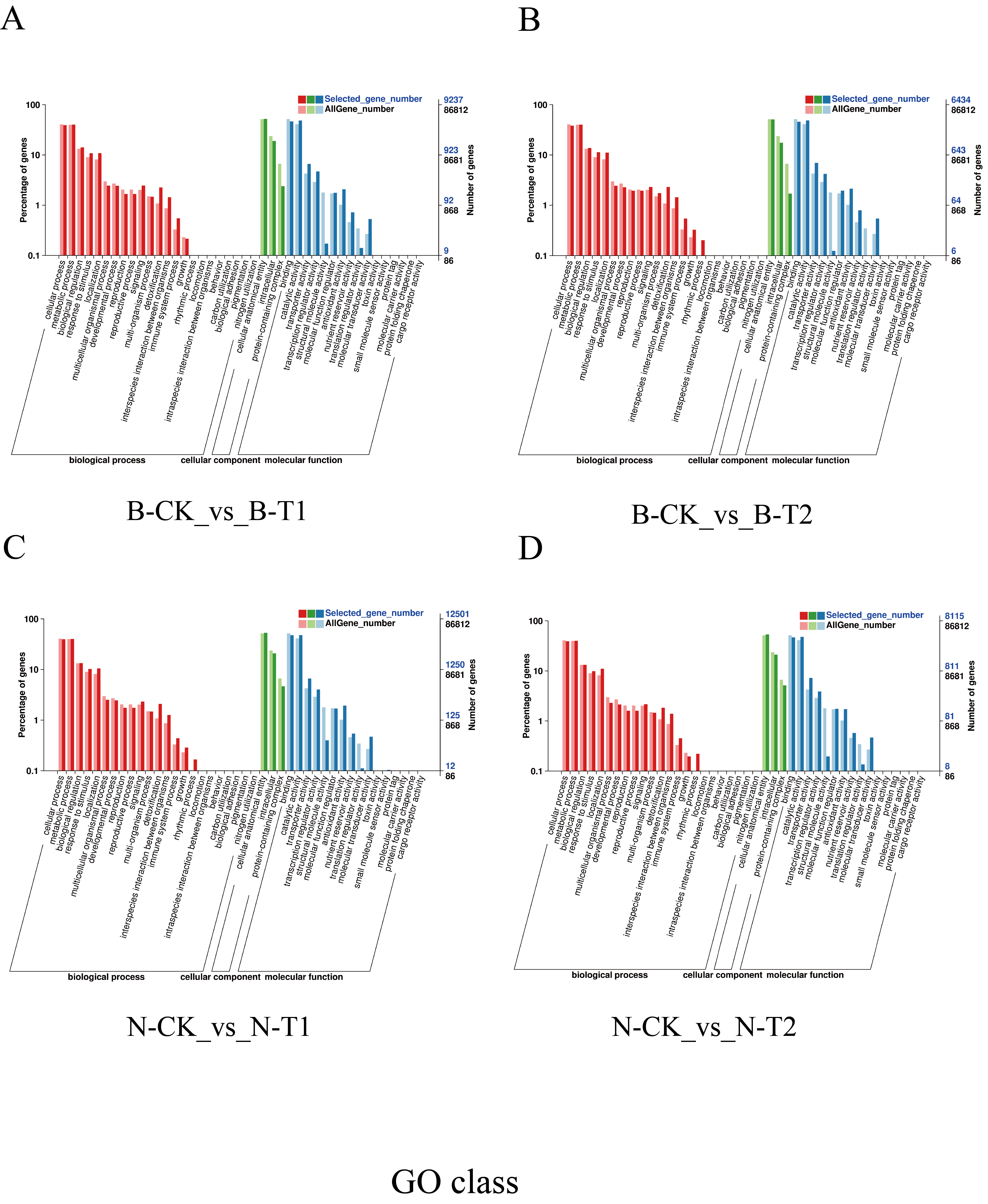

Supplement: Supplementary file 1 [file ijms-26-00331-s001.zip › Supplemental Figure S1.png]

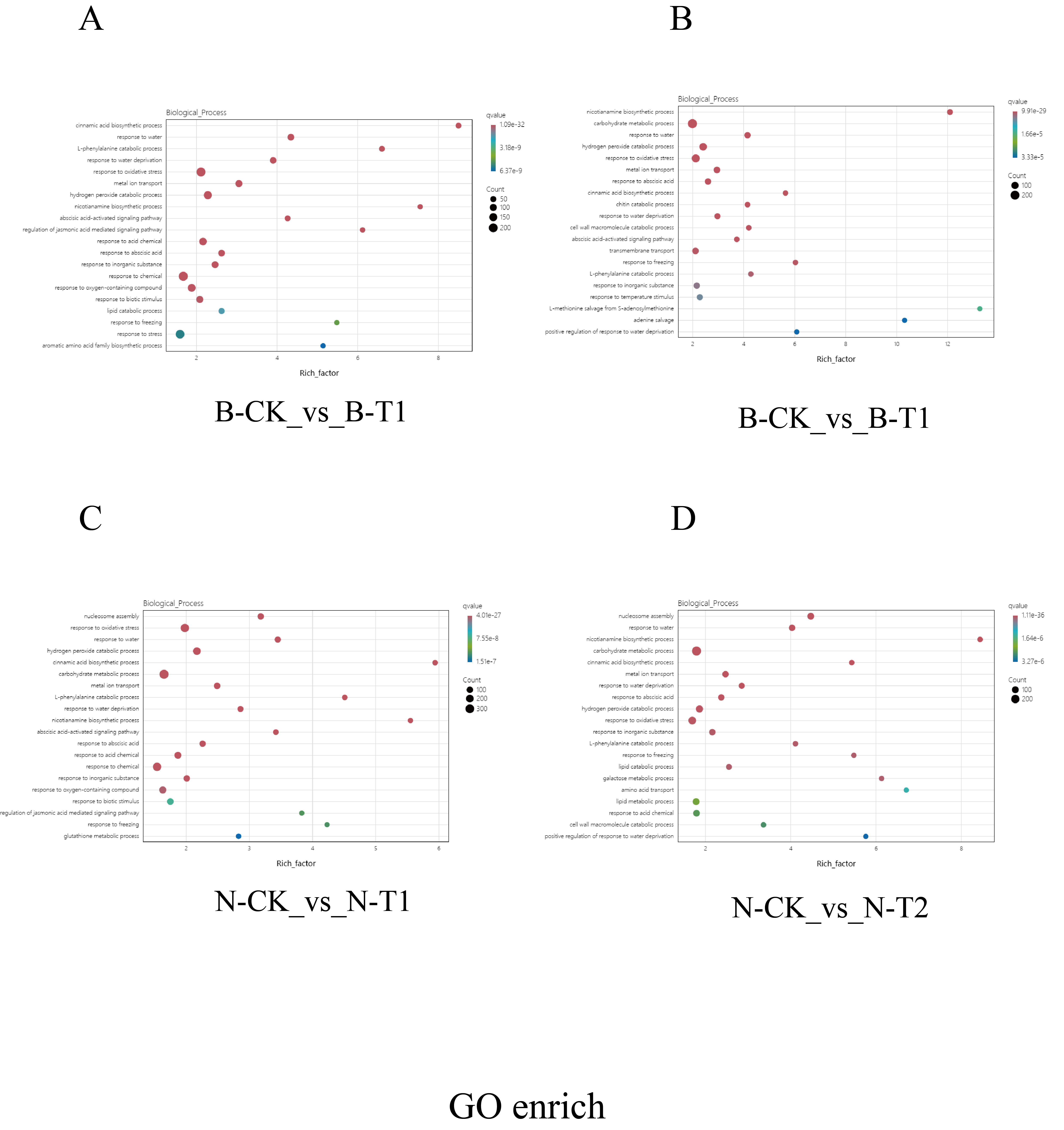

Supplement: Supplementary file 1 [file ijms-26-00331-s001.zip › Supplemental Figure S2.png]

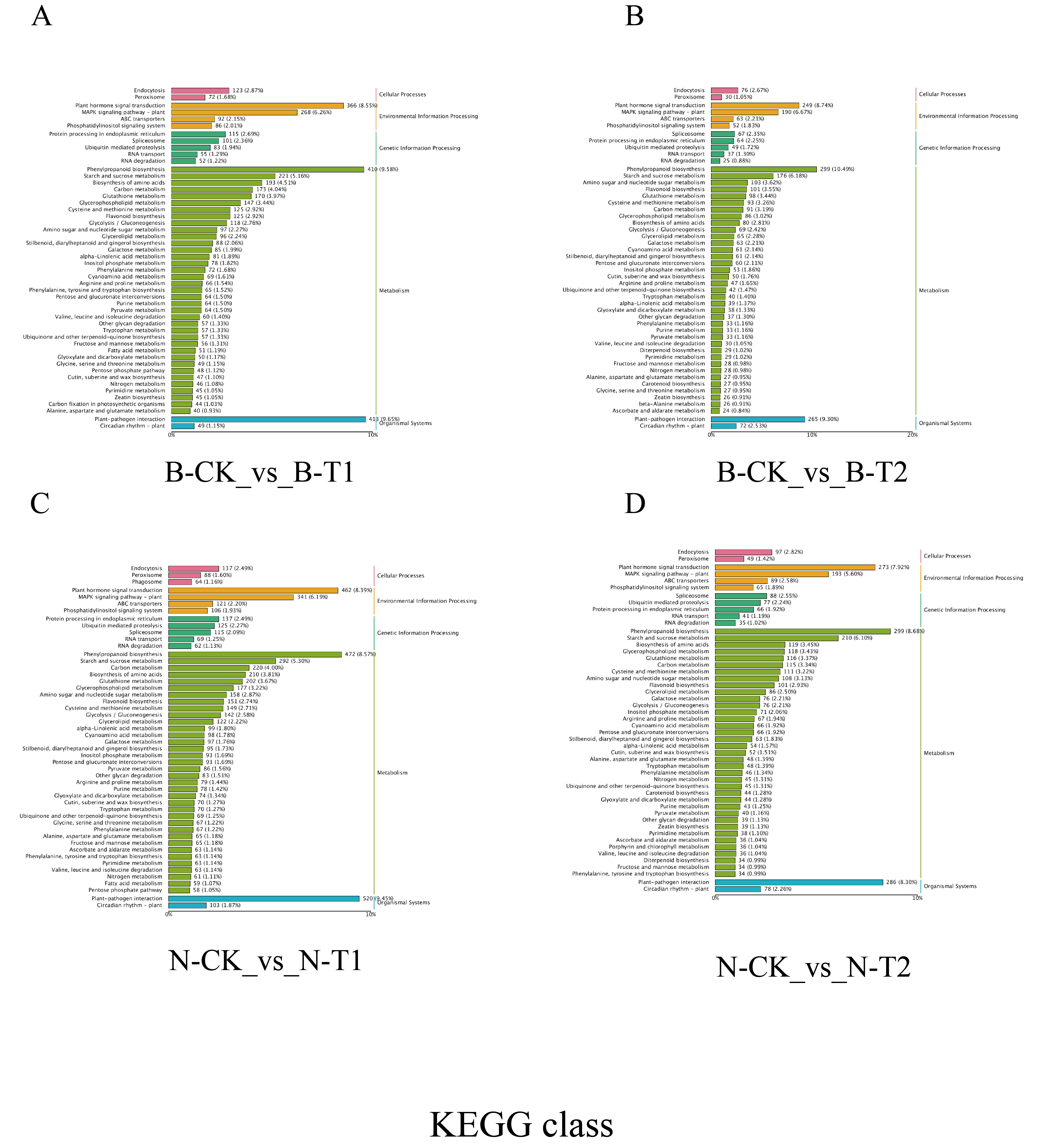

Supplement: Supplementary file 1 [file ijms-26-00331-s001.zip › Supplemental Figure S3.png]

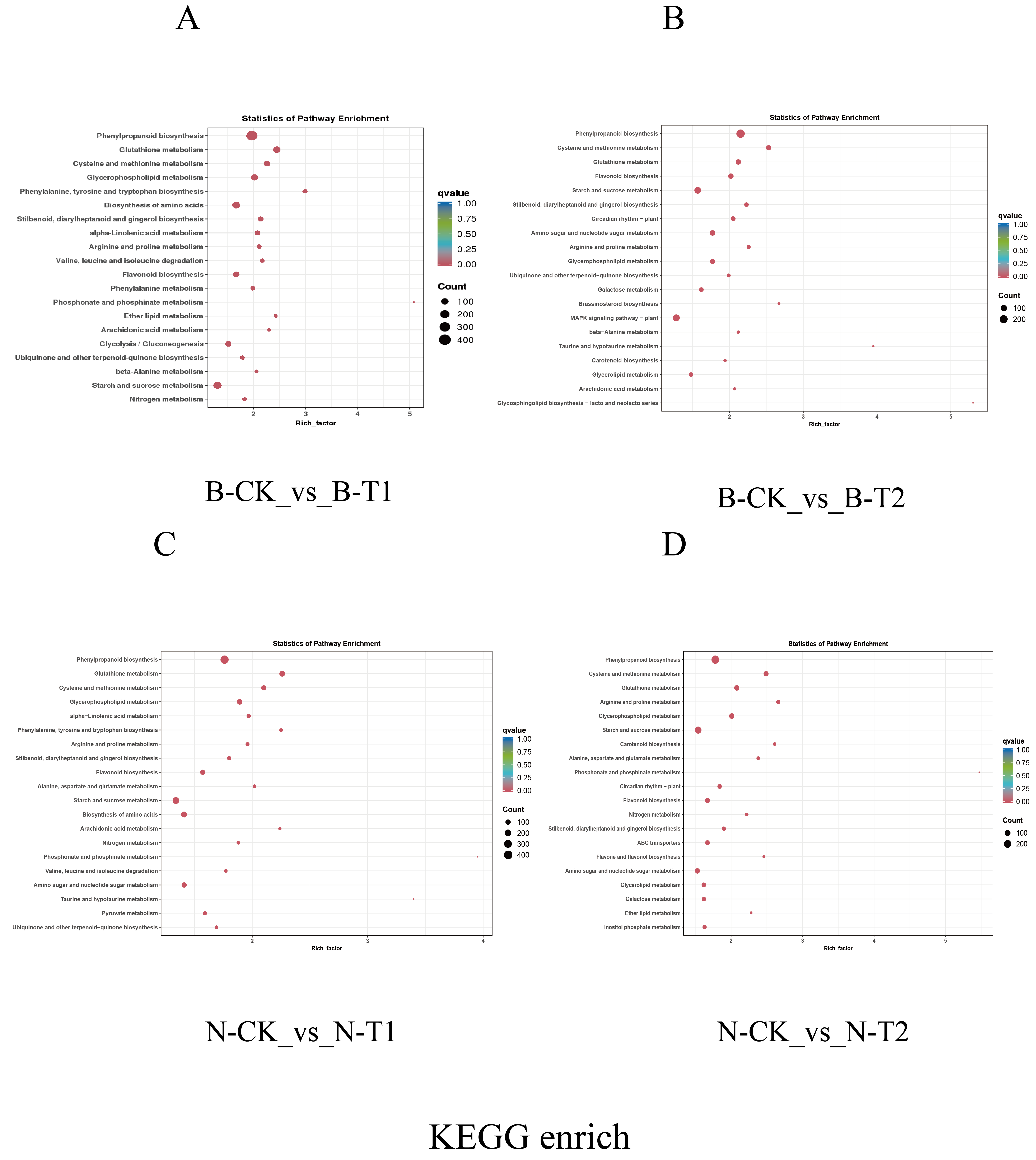

Supplement: Supplementary file 1 [file ijms-26-00331-s001.zip › Supplemental Figure S4.png]
